# Ovate Pontic Design in the Esthetic Replacement of Maxillary Lateral Incisors: A Case Report

**DOI:** 10.7759/cureus.114175

**Published:** 2026-08-08

**Authors:** Asha Rathod, Gaurang Mistry, Rajeev Singh, Mansi Shukla, Foram Dama, Sanpreet S Sachdev

**Affiliations:** 1 Prosthodontics, DY Patil University School of Dentistry, Navi Mumbai, IND; 2 Oral Pathology and Microbiology, Bharati Vidyapeeth (Deemed to be University) Dental College and Hospital, Navi Mumbai, IND

**Keywords:** anterior esthetics, crown lengthening, fixed dental prosthesis, ovate pontic, zirconia

## Abstract

Esthetic replacement of missing teeth in the anterior maxilla requires coordinated management of gingival contours, pontic emergence profiles, restorative materials, and occlusal function. This case report describes the interdisciplinary rehabilitation of a 21-year-old female patient whose maxillary lateral incisors had been extracted following failed endodontic treatment. Clinical examination revealed a high smile line with approximately 4 mm of gingival display and bilateral Siebert Class I ridge defects at the healed extraction sites. Treatment comprised diode laser-assisted esthetic gingival recontouring, intentional endodontic treatment of the selected abutments, and progressive development of the ovate pontic sites using a provisional restoration over 6-8 weeks. A six-unit monolithic zirconia fixed dental prosthesis extending from 13 to 23 was subsequently fabricated and cemented. Occlusal adjustment maintained central-incisor guidance during protrusion and canine guidance during lateral excursions, while the ovate pontics remained free of centric and excursive contacts. At the one- and three-month follow-up visits, the peri-pontic tissues demonstrated stable contours, minimal plaque accumulation, and no bleeding, ulceration, debonding, or fracture. The patient reported satisfactory esthetics, phonetics, mastication, and comfort. Longer follow-up is required to confirm the stability of the soft tissues and prosthesis.

## Introduction

Esthetic rehabilitation of the anterior maxilla requires coordinated consideration of facial appearance, gingival architecture, tooth proportions, phonetics, and occlusal function. Minor discrepancies in this highly visible region are readily perceived and may considerably influence smile attractiveness [1]. An esthetically pleasing result therefore depends on the relationship between the upper lip, gingival display, incisal edges, gingival zeniths, interdental papillae, and the apparent emergence of the teeth from the surrounding soft tissues [2].

Excessive gingival display may result from a high smile line, altered passive eruption, vertical maxillary excess, dentoalveolar extrusion, upper-lip hypermobility, short clinical crowns, or a combination of these factors [3]. Treatment must therefore be selected according to the underlying cause and may involve periodontal, orthodontic, surgical, or restorative approaches. When unfavorable gingival contours or inadequate clinical crown proportions compromise the planned restoration, conservative gingival recontouring can establish a more harmonious soft-tissue framework [4]. Such periodontal procedures should be coordinated with prosthodontic planning because alterations in gingival margins can affect papillary form, tissue stability, and the final restorative outcome [5].

Pontic design is an important determinant of the biological, functional, and esthetic performance of a fixed dental prosthesis. The principal conventional designs include hygienic, conical, saddle or full ridge-lap, modified ridge-lap, and ovate pontics. A hygienic pontic has no contact with the residual ridge and provides favorable access for cleaning but is generally restricted to non-esthetic posterior regions. A conical pontic provides limited tissue contact and is more suitable for thin, rounded posterior ridges. Although a full ridge-lap pontic resembles the contours of a natural tooth, its extensive facial and lingual tissue contact can interfere with plaque control. The modified ridge-lap design combines facial ridge overlap with reduced lingual contact and therefore offers a balance between esthetics and cleansability [6].

The ovate pontic differs from these designs because its smooth, convex cervical portion extends into a prepared soft-tissue concavity. This configuration can create the appearance of a natural tooth emerging from the gingiva while supporting the cervical contour and surrounding papillary tissues. Its use is particularly advantageous in the anterior esthetic region; however, the outcome depends on residual ridge anatomy, adequate soft-tissue volume, controlled tissue pressure, and meticulous oral hygiene. In healed extraction sites with loss of soft-tissue contour, progressive conditioning with a provisional restoration can develop the recipient site and refine the emergence profile before fabrication of the definitive prosthesis [6].

Material selection must complement the pontic design and provide sufficient strength, marginal accuracy, biocompatibility, and esthetic integration. All-ceramic fixed dental prostheses have demonstrated acceptable clinical outcomes when used with appropriate case selection and prosthetic design [7]. Zirconia provides favorable mechanical properties for multi-unit restorations and can be digitally designed to obtain suitable connector dimensions and pontic contours. Monolithic zirconia additionally permits the fabrication of the prosthesis from a single ceramic material, while appropriate staining, glazing, and surface characterization can assist its integration with the adjacent anterior dentition [8].

This case report describes the interdisciplinary rehabilitation of bilaterally missing maxillary lateral incisors associated with a high smile line and horizontal ridge deficiency. Treatment involved diode laser-assisted esthetic gingival recontouring, progressive development of ovate pontic sites, and rehabilitation with a six-unit monolithic zirconia fixed dental prosthesis.

## Case presentation

Patient information and clinical findings

A 21-year-old female patient reported to the Department of Prosthodontics with the chief complaint of missing upper anterior teeth, excessive gingival display during smiling, and dissatisfaction with her dental appearance. She reported increasing self-consciousness during social interactions and desired a fixed restoration that would resemble the natural dentition.

The patient's medical history was non-contributory, with no systemic illness, drug allergy, or contraindication to periodontal or prosthodontic treatment. The maxillary right and left lateral incisors (12 and 22) had previously been extracted following failed endodontic treatment and were considered to have a poor long-term prognosis. The healed extraction sites subsequently demonstrated loss of facial soft-tissue contour, which compromised the emergence profile in the lateral incisor regions. The patient maintained satisfactory oral hygiene and reported no parafunctional habits, including bruxism, nail biting, or tongue thrusting.

Extraoral examination revealed a high smile line with approximately 4 mm of gingival display during smiling. Intraoral examination demonstrated missing maxillary lateral incisors and altered gingival contours in the anterior maxilla. The patient had an overbite of 3 mm, corresponding to approximately 30% overlap of the mandibular incisors, and an overjet of 2 mm. Periodontal examination demonstrated probing depths not exceeding 3 mm, absence of bleeding on probing, and no clinical attachment loss. The edentulous sites at 12 and 22 demonstrated Siebert Class I ridge defects, characterized by horizontal tissue deficiency with preservation of ridge height. No clinical evidence of altered passive eruption was observed, and the excessive gingival display was attributed predominantly to the high smile line.

The facial and dental midlines were clinically acceptable, and the remaining maxillary anterior teeth demonstrated adequate periodontal support. Occlusal examination showed protrusive guidance predominantly through the maxillary and mandibular central incisors. Lateral excursions demonstrated canine-guided occlusion, with no non-working-side interferences. Preoperative extraoral and intraoral photographs were obtained.

**Figure 1:**
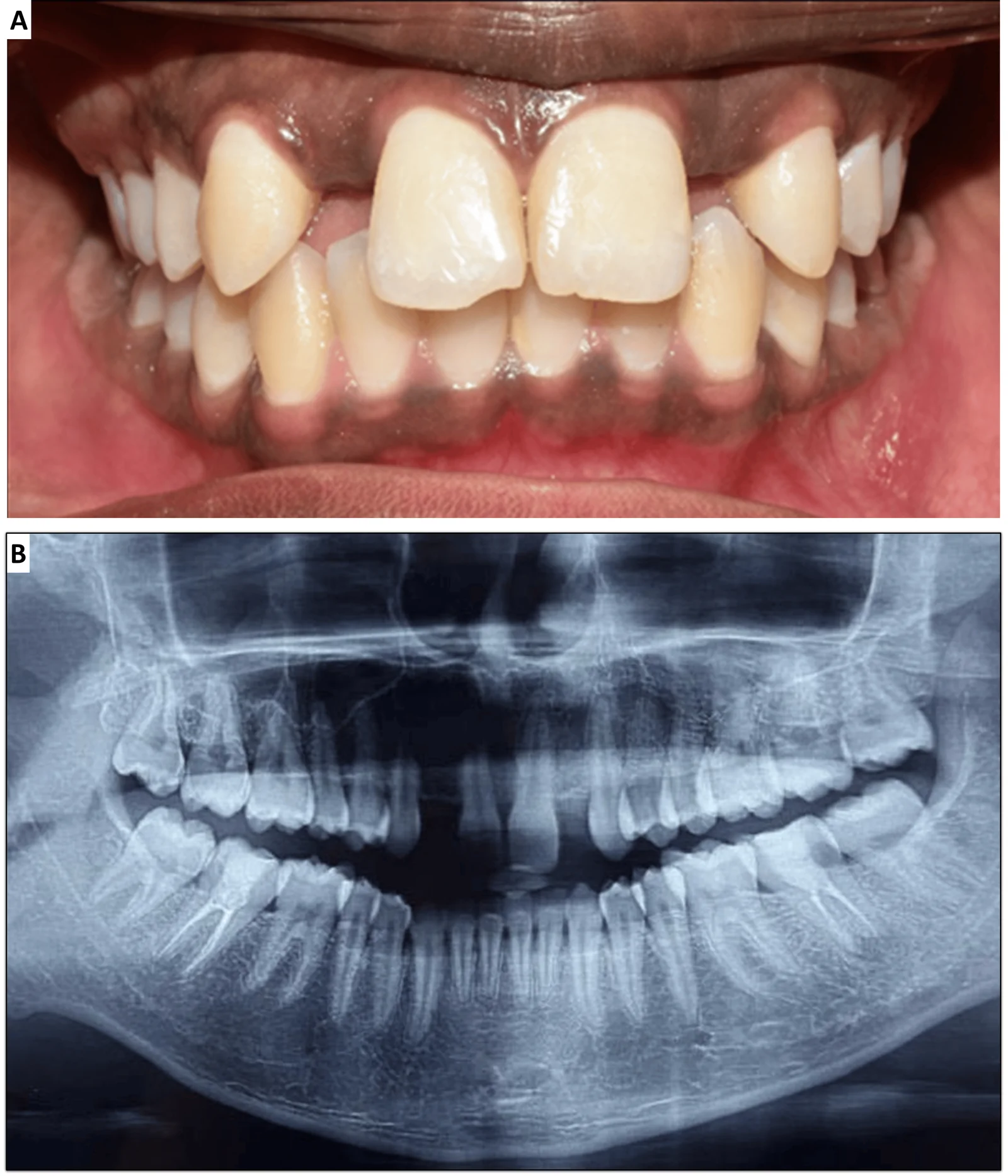
(A) Preoperative frontal intraoral view showing the missing maxillary lateral incisors and (B) orthopantomogram showing the healed extraction sites and supporting structures of the proposed abutment teeth

A preoperative orthopantomogram was obtained as part of the comprehensive diagnostic assessment. The orthopantomogram was selected because treatment planning involved multiple anterior abutment teeth and required simultaneous evaluation of the overall dentition, root morphology, alveolar bone support, healed extraction sites, and adjacent anatomical structures. It demonstrated absence of 12 and 22, satisfactory bone support around the proposed abutment teeth, and no evident periapical or osseous abnormalities. The panoramic findings were not interpreted in isolation but were correlated with the clinical, periodontal, and occlusal examinations. Based on these collective findings, the proposed abutment teeth were considered suitable for the planned fixed dental prosthesis (Figure 1). The final diagnosis was bilateral partial edentulism in the maxillary lateral incisor regions associated with Siebert Class I ridge defects, altered anterior gingival contours, and excessive gingival display related to a high smile line.

Diagnostic assessment and treatment planning

Diagnostic impressions were made, and casts were prepared. A diagnostic wax-up was completed to evaluate tooth proportions, available restorative space, gingival harmony, pontic emergence profiles, and the anticipated esthetic outcome. The treatment objectives were to replace the missing maxillary lateral incisors, harmonize the anterior gingival contours, develop natural emergence profiles at the pontic sites, and restore esthetics and function with a fixed dental prosthesis.

Different treatment alternatives were discussed with the patient. Implant-supported single crowns were considered; however, the patient wished to avoid additional implant surgery, increased treatment duration, and greater financial expenditure. Resin-bonded fixed dental prostheses were discussed as a conservative alternative. However, this option was not selected because correction of the abutment contours and axial inclinations, coordinated management of the bilateral edentulous spaces, and controlled development of the ovate pontic sites were required. Orthodontic space redistribution followed by definitive prosthetic rehabilitation was also discussed, but the patient declined orthodontic treatment because of the longer treatment duration. The option of no treatment was also discussed; however, it would not have addressed the patient's esthetic concerns.

Considering the bilateral absence of the maxillary lateral incisors, horizontal ridge deficiency, altered gingival contours, abutment inclinations, and the patient's preference for a fixed restoration, an interdisciplinary treatment plan comprising diode laser-assisted esthetic gingival recontouring, intentional endodontic treatment of the selected abutments, progressive development of ovate pontic sites, and rehabilitation with a six-unit monolithic zirconia fixed dental prosthesis was selected. Written informed consent was obtained for the proposed treatment and publication of the anonymized clinical information and images.

Periodontal management

Following the completion of the diagnostic wax-up, a surgical guide was fabricated to transfer the planned gingival contours to the clinical situation. After the administration of local anesthesia, conservative esthetic gingival recontouring was performed using a diode laser. Excess gingival tissue was excised according to the surgical guide to improve the symmetry of the gingival margins and apparent clinical crown proportions while preserving a harmonious anterior gingival architecture [4,5]. Hemostasis was achieved with the diode laser, and suturing was not required.

Postoperative instructions and analgesics were prescribed, together with a 0.12% chlorhexidine mouth rinse. The patient was reviewed after one week, at which time healing was uneventful. A healing period of approximately eight weeks was allowed for soft-tissue maturation before the commencement of definitive prosthodontic treatment.

Endodontic and abutment management

The maxillary central incisors and canines (11, 13, 21, and 23) selected as abutments were asymptomatic and demonstrated no clinical or evident panoramic radiographic signs of periapical pathology. Intentional endodontic treatment was performed to facilitate the extent of tooth preparation required, correct unfavorable axial inclinations, create sufficient restorative space, and establish a common path of insertion for the planned six-unit fixed dental prosthesis. Following the completion of endodontic treatment, the teeth were evaluated and considered suitable for use as abutments.

Prosthodontic procedures

Following periodontal healing, the maxillary central incisors and canines were prepared as retainers for the fixed dental prosthesis. A circumferential deep chamfer finish line with rounded internal line angles was prepared. An axial reduction of approximately 1.5 mm and an incisal reduction of approximately 2 mm were provided to obtain adequate restorative space [6].

A provisional polymethyl methacrylate fixed dental prosthesis was fabricated. The provisional restoration extended from tooth 13 to tooth 23, with 11, 13, 21, and 23 serving as retainers and 12 and 22 as ovate pontics. The tissue-contacting surfaces of the provisional pontics were finished with smooth, convex contours and extended approximately 1-1.5 mm into the soft tissues at the healed extraction sites. This produced controlled tissue displacement without persistent blanching, ischemia, or ulceration. The provisional restoration was relined with composite resin in the pontic regions and cemented with temporary luting material (OraTemp, Prevest DenPro Ltd., Jammu, India).

**Figure 2:**
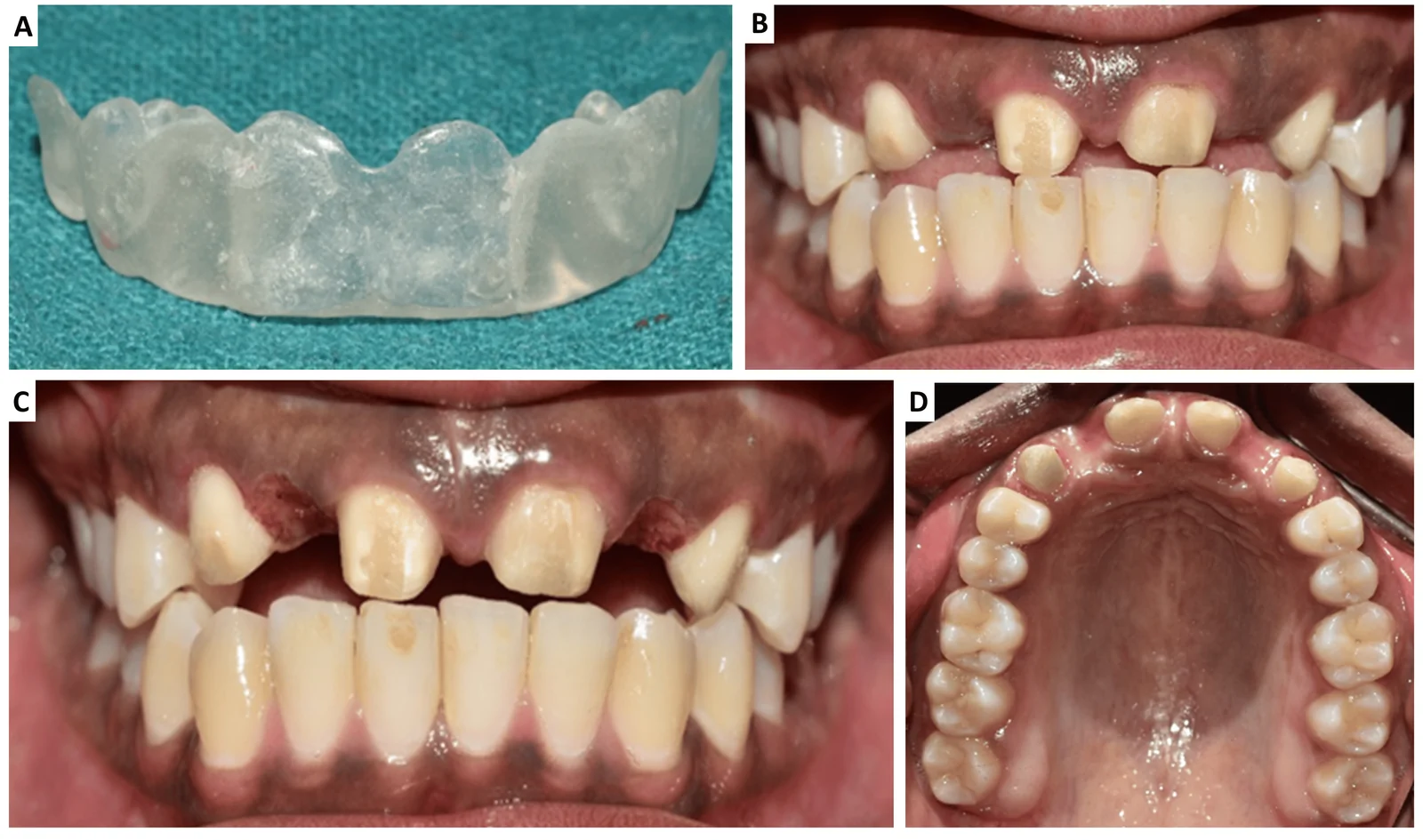
(A) Surgical guide used for gingival recontouring, (B) diode laser-assisted esthetic gingival recontouring, (C) progressive conditioning of the ovate pontic recipient sites, and (D) healed and conditioned pontic sites

The patient was reviewed at two-week intervals, and incremental modifications were made to the cervical contours of the provisional pontics. This progressive conditioning guided the tissues into stable concavities and refined the cervical emergence profiles [6]. Soft-tissue conditioning was continued for approximately 6-8 weeks. At the end of this period, the peri-pontic tissues were healthy, and stable recipient sites had been established for the definitive ovate pontics (Figure 2).

After satisfactory soft-tissue conditioning, gingival displacement was achieved using braided retraction cords. The definitive impression was recorded using a two-step putty-wash technique with an addition silicone impression material (Hydrorise, Zhermack SpA, Badia Polesine, Italy). Care was taken to reproduce the preparation finish lines and conditioned ovate pontic recipient sites accurately [6].

The definitive prosthesis was digitally designed using dental computer-aided design software (exocad DentalCAD, exocad GmbH, Darmstadt, Germany). A full-contour six-unit monolithic zirconia fixed dental prosthesis extending from 13 to 23 was designed, with 11, 13, 21, and 23 as retainers and 12 and 22 as ovate pontics. The prosthesis was milled from a zirconia blank (Upcera, Shenzhen Upcera Dental Technology Co. Ltd., Shenzhen, China) using a milling unit (imes-icore GmbH, Eiterfeld, Germany). It was subsequently sintered in a TABEO sintering furnace (MIHM-VOGT GmbH & Co. KG, Stutensee, Germany) according to the recommended protocol. Connector dimensions were maintained at approximately 9 mm² to provide adequate mechanical strength while preserving acceptable anterior contours [8].

Shade selection was performed under natural daylight using the VITA Classical shade guide (VITA Zahnfabrik, Bad Säckingen, Germany), and shade A2 was selected. The monolithic zirconia prosthesis was characterized and finished to reproduce the selected shade, surface texture, and contours of the adjacent dentition.

**Figure 3:**
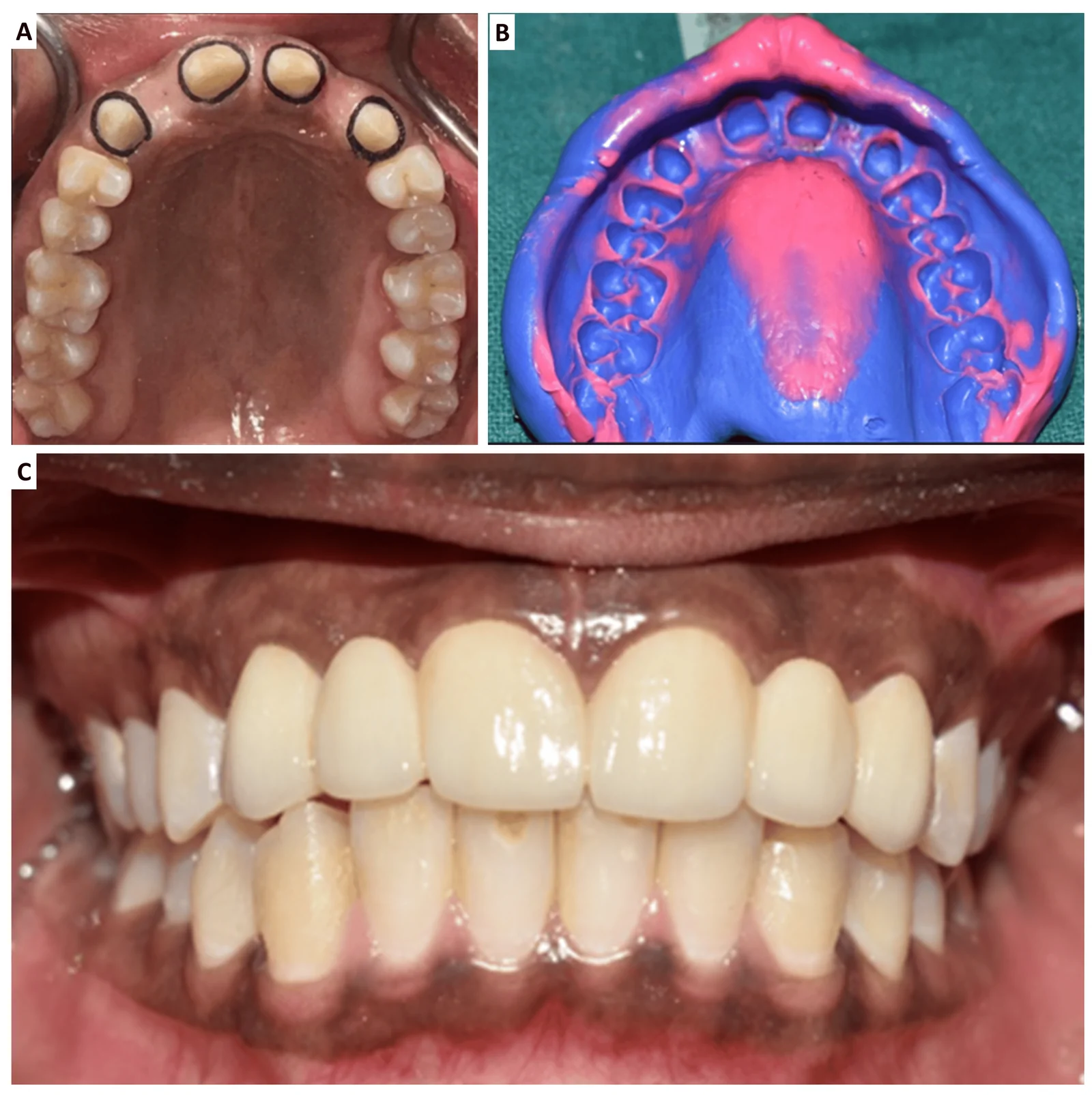
(A) Gingival displacement using retraction cords, (B) definitive impression, and (C) definitive six-unit monolithic zirconia fixed dental prosthesis with ovate pontics

During clinical evaluation, the definitive prosthesis was assessed for marginal adaptation, proximal contacts, shade match, contour, pontic emergence profile, gingival integration, phonetics, and patient acceptance. After satisfactory evaluation, the prosthesis was definitively luted using a self-adhesive dual-cure resin cement (RelyX U200, 3M ESPE, St. Paul, Minnesota, United States) under adequate isolation. Excess cement was carefully removed, and the cervical surfaces were evaluated for gingival adaptation and accessibility for oral hygiene (Figure 3).

Occlusal management

Following cementation, the prosthesis was adjusted to establish stable, bilaterally distributed centric contacts on the retainer teeth. Protrusive guidance was provided predominantly by the maxillary and mandibular central incisors, while lateral excursions were guided by the canines. Working- and non-working-side interferences were eliminated. The ovate pontics at 12 and 22 were maintained free of centric and excursive contacts to avoid transmitting undesirable functional forces to the conditioned pontic sites.

Follow-up and clinical outcome

**Figure 4:**
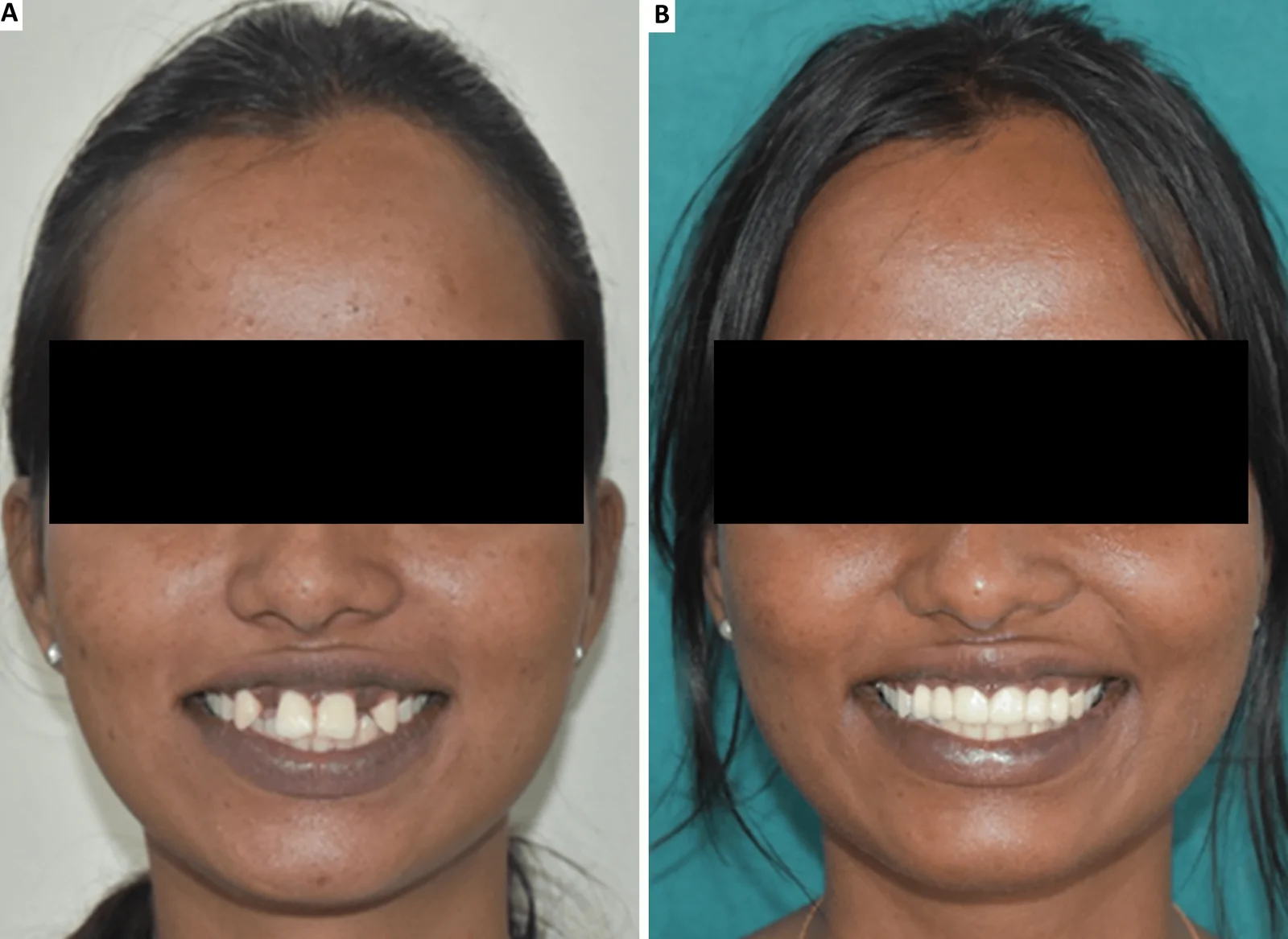
Comparison of the front facial profile for (A) preoperative and (B) postoperative smiles

The postoperative smile demonstrated improved gingival harmony, replacement of the missing maxillary lateral incisors, and satisfactory integration of the prosthesis with the surrounding soft tissues (Figure 4). The patient was reviewed at one month and three months after definitive cementation.

At both follow-up visits, the peri-pontic tissues demonstrated stable architecture, minimal plaque accumulation, absence of bleeding on probing, and no tissue ulceration. The prosthesis exhibited satisfactory marginal adaptation and remained stable, with no evidence of debonding or fracture. No food lodgment, phonetic difficulty, or patient discomfort was reported. The patient expressed excellent satisfaction with the esthetics, phonetics, mastication, and overall comfort of the restoration.

The patient was instructed to clean beneath the ovate pontics using interdental cleaning aids and to maintain regular professional recall visits. Continued follow-up was advised to monitor peri-pontic tissue health, marginal integrity, occlusal stability, and the long-term performance of the prosthesis.

## Discussion

The present case demonstrates the importance of coordinating periodontal and prosthodontic procedures when missing anterior teeth are accompanied by unfavorable gingival display and loss of ridge contour. The principal challenge was not limited to replacing the maxillary lateral incisors. A natural result required harmonization of the gingival margins, development of suitable pontic recipient sites, restoration of appropriate tooth proportions, and maintenance of a functional occlusal scheme. The high smile line and approximately 4 mm of gingival display increased the visibility of minor discrepancies in pontic contour and gingival symmetry. Therefore, the soft-tissue and prosthetic components were planned together rather than managed as separate procedures [4,5].

Excessive gingival display may arise from altered passive eruption, vertical maxillary excess, dentoalveolar extrusion, upper-lip hypermobility, a high smile line, or a combination of these factors [3]. In the present patient, no clinical evidence of altered passive eruption was identified, and the gingival display was attributed predominantly to the high smile line. Diode laser-assisted gingival recontouring was therefore performed conservatively to improve gingival symmetry and clinical crown proportions rather than to correct an osseous abnormality. The surgical guide assisted in transferring the planned gingival levels to the clinical situation, while the eight-week healing period allowed adequate soft-tissue maturation before definitive treatment. Careful case selection and preservation of the supracrestal tissue attachment are important because excessive tissue removal can produce gingival rebound, attachment loss, or unstable restorative margins [4,5].

Although panoramic radiography provides less fine detail than intraoral periapical imaging, it remained useful because the rehabilitation involved four anterior abutments, two healed extraction sites, and assessment of the entire dentition. The orthopantomogram permitted a comprehensive survey of root morphology, alveolar support, edentulous sites, and adjacent structures. It was interpreted with the clinical, periodontal, and occlusal findings rather than used alone to diagnose subtle localized endodontic changes.

Pontic design was selected according to the anterior location of the edentulous spaces and the morphology of the residual ridges. Hygienic and conical pontics were unsuitable because they could not reproduce a natural cervical emergence profile in the maxillary anterior region. A full ridge-lap pontic would have produced extensive tissue contact and restricted access for plaque control, whereas a modified ridge-lap design would not have adequately corrected the loss of cervical tissue contour. The edentulous regions demonstrated Siebert Class I defects, characterized by horizontal tissue loss with preservation of ridge height. This anatomy permitted the development of soft-tissue concavities for ovate pontics without the need for vertical ridge augmentation. The ovate design was therefore selected to create the appearance of the lateral incisors emerging naturally from the gingiva while maintaining smooth and cleansable tissue-contacting surfaces [6].

Progressive provisionalization was central to developing the healed extraction sites because they initially lacked the contours required for definitive ovate pontics. Controlled extension of the provisional pontics into the soft tissues allowed the gradual development of the recipient sites without persistent blanching, ischemia, or ulceration. Adjustments at two-week intervals permitted the incremental modification of the emergence profile instead of attempting complete tissue displacement during a single appointment. Choi et al. described the preservation of pontic-site tissue volume using the root-submergence technique and emphasized the importance of maintaining ridge form in the maxillary esthetic region [9]. Iida et al. similarly demonstrated that ovate pontic provisionalization could assist in preserving peri-pontic architecture before delayed implant placement [10]. Although neither root submergence nor an implant pathway was used in the present case, these reports support the shared principle that controlled provisionalization can preserve or guide the soft-tissue form required for an esthetic restoration.

Implant-supported crowns and resin-bonded prostheses would ordinarily be considered for replacing missing maxillary lateral incisors in a young patient. Implant therapy was declined because the patient wished to avoid additional surgery, increased treatment duration, and greater financial expenditure. Blatz et al. described anterior cantilever zirconia resin-bonded fixed dental prostheses with soft-tissue augmentation as a conservative option for lateral incisor replacement [11]. In the present case, a conventional six-unit fixed dental prosthesis was selected because bilateral replacement, correction of unfavorable abutment inclinations, and coordinated development of two pontic sites were required. Nevertheless, intentional endodontic treatment and full-coverage preparation of four asymptomatic teeth represent biologically invasive components of treatment. This approach should not be considered routine and must remain limited to carefully selected situations after conservative, orthodontic, implant-supported, and resin-bonded alternatives have been discussed.

The definitive restoration was fabricated as a six-unit monolithic zirconia fixed dental prosthesis rather than a feldspathic porcelain-veneered zirconia restoration. Zirconia provides favorable mechanical properties for multi-unit prostheses, while computer-aided design and manufacturing permit control over connector dimensions, marginal form, and pontic contours [7,8]. A monolithic design eliminates the need for a separate veneering porcelain layer and removes the veneer-framework interface as a potential site of chipping. However, reproducing the translucency and optical characteristics of natural anterior teeth may be more challenging with monolithic zirconia, making appropriate material selection, staining, glazing, and surface characterization important. Long-term evidence for anterior zirconia fixed dental prostheses has predominantly involved veneered designs. Solá-Ruiz et al. reported acceptable outcomes after seven years [12], while their 12-year evaluation identified veneer chipping and biological complications as continuing concerns [13]. These findings support continued clinical monitoring even when a monolithic design is used.

Occlusal management was important because the restoration replaced two anterior teeth as part of a multi-unit prosthesis. The pretreatment occlusion showed central-incisor guidance during protrusion, canine guidance during lateral excursions, and no non-working-side interferences. These relationships were maintained after definitive cementation. Centric contacts were distributed over the retainer teeth, while the ovate pontics were kept free of centric and excursive contacts. This arrangement minimized the transmission of undesirable functional forces to the pontic sites and avoided introducing new excursive interferences. Periodic assessment remains necessary because occlusal wear, minor tooth movement, or changes in prosthesis integrity may alter force distribution over time [6].

At the one- and three-month follow-up visits, the peri-pontic tissues demonstrated stable contours, minimal plaque accumulation, absence of bleeding on probing, and no ulceration. The prosthesis retained satisfactory marginal adaptation without debonding or fracture, and the patient reported favorable esthetics, phonetics, mastication, and comfort. These findings represent a satisfactory early clinical outcome but cannot establish long-term tissue stability or prosthesis survival.

The principal limitation of this case report is the short follow-up period of three months. Additional limitations include the absence of standardized esthetic indices, quantitative three-dimensional evaluation of soft-tissue changes, and validated patient-reported outcome measures. As a single case, the findings cannot establish the comparative effectiveness of ovate pontics or monolithic zirconia restorations. Follow-up at six and 12 months and annually thereafter should include the assessment of plaque accumulation, bleeding on probing, probing depths, papillary fill, stability of the pontic-site contours, marginal integrity, occlusal contacts, and prosthesis survival. Future case series incorporating standardized photographs, digital soft-tissue scans, and validated satisfaction scales would provide more objective evidence regarding the predictability of this treatment approach.

## Conclusions

A coordinated periodontal-prosthodontic approach combining conservative gingival recontouring and progressive ovate pontic site conditioning provided a natural emergence profile for the replacement of bilaterally missing maxillary lateral incisors. The six-unit monolithic zirconia fixed dental prosthesis demonstrated satisfactory esthetic and functional integration, with healthy peri-pontic tissues and no biological or mechanical complications at three months. Longer follow-up is required to confirm the stability of the soft-tissue contours and prosthesis.

## References

[1] Parrini S, Rossini G, Castroflorio T, Fortini A, Deregibus A, Debernardi C (2016). Laypeople's perceptions of frontal smile esthetics: a systematic review. Am J Orthod Dentofacial Orthop.

[2] Omar D, Duarte C (2018). The application of parameters for comprehensive smile esthetics by digital smile design programs: a review of literature. Saudi Dent J.

[3] Dym H, Pierre R 2nd (2020). Diagnosis and treatment approaches to a "gummy smile". Dent Clin North Am.

[4] Al-Harbi F, Ahmad I (2018). A guide to minimally invasive crown lengthening and tooth preparation for rehabilitating pink and white aesthetics. Br Dent J.

[5] Pilalas I, Tsalikis L, Tatakis DN (2016). Pre-restorative crown lengthening surgery outcomes: a systematic review. J Clin Periodontol.

[6] Rosenstiel SF, Land MF, Fujimoto J (2015). Contemporary Fixed Prosthodontics. https://books.google.co.in/books?hl=en&lr=&id=BKh2EAAAQBAJ&oi=fnd&pg=PP1&dq=Contemporary+Fixed+Prosthodontics&ots=Qw9jNXTt8T&sig=YcAHHN74aNETKzqB25csmJeHZdo&redir_esc=y#v=onepage&q=Contemporary%20Fixed%20Prosthodontics&f=false.

[7] Pjetursson BE, Sailer I, Makarov NA, Zwahlen M, Thoma DS (2015). All-ceramic or metal-ceramic tooth-supported fixed dental prostheses (FDPs)? A systematic review of the survival and complication rates. Part II: multiple-unit FDPs. Dent Mater.

[8] Le M, Papia E, Larsson C (2015). The clinical success of tooth- and implant-supported zirconia-based fixed dental prostheses. A systematic review. J Oral Rehabil.

[9] Choi S, Yeo IS, Kim SH, Lee JB, Cheong CW, Han JS (2015). A root submergence technique for pontic site development in fixed dental prostheses in the maxillary anterior esthetic zone. J Periodontal Implant Sci.

[10] Iida Y, Sheng S, Tsukiboshi Y, Min S (2023). Ridge preservation with the socket-shield technique and immediate provisionalization for delayed implant placement. A case report. Int J Esthet Dent.

[11] Blatz MB, Rotondo T, Hant S, Prott LS (2026). Optimizing hard and soft-tissue esthetics with anterior cantilever zirconia ceramic resin-bonded fixed dental prostheses. J Esthet Restor Dent.

[12] Solá-Ruíz MF, Agustin-Panadero R, Fons-Font A, Labaig-Rueda C (2015). A prospective evaluation of zirconia anterior partial fixed dental prostheses: clinical results after seven years. J Prosthet Dent.

[13] Solá-Ruiz MF, Leon-Martine R, Labaig-Rueda C, Selva-Otalaorrouchi E, Agustín-Panadero R (2022). Clinical outcomes of veneered zirconia anterior partial fixed dental prostheses: a 12-year prospective clinical trial. J Prosthet Dent.

